# 2-Methyl-3-nitro­benzyl cyanide

**DOI:** 10.1107/S1600536809008484

**Published:** 2009-03-14

**Authors:** You-Sheng Chen, Jian-Hong Zhang

**Affiliations:** a Department of Pharmacy, GuangDong Vocational and Technical College of Chemical Engineering Pharmaceutics, Guangzhou 510520, People’s Republic of China; b Department of Applied Chemistry, College of Science, Nanjing University of Technology, Nanjing 210009, People’s Republic of China

## Abstract

The title compound, C_9_H_8_N_2_O, was prepared from *o*-xylene by nitration, oxidation, hydrolysis, reduction, chlorination and cyanation. There are two mol­ecules in the asymmetric unit with a dihedral angle of 20.15 (7)° between their aromatic rings.

## Related literature

For related literature, see: Wang *et al.* (1999 ▶).
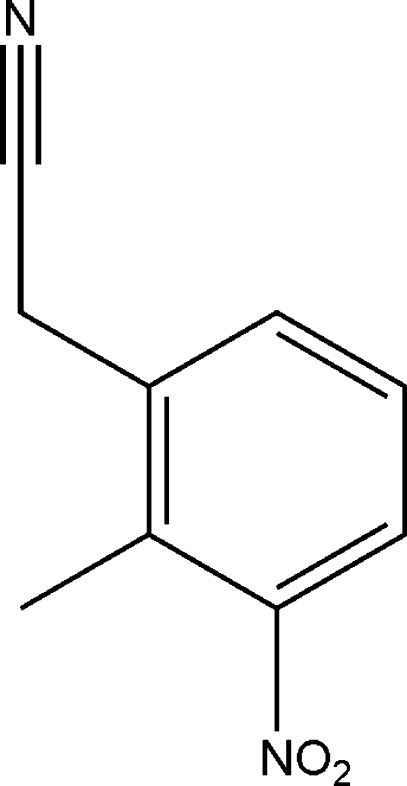


## Experimental

### 

#### Crystal data

C_9_H_8_N_2_O_2_
*M* *_r_* = 176.18
Monoclinic, 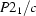

*a* = 17.216 (3) Å
*b* = 7.1950 (14) Å
*c* = 15.746 (3) Å
β = 117.10 (3)°
*V* = 1736.3 (7) Å^3^
*Z* = 8
Mo *K*α radiation
μ = 0.10 mm^−1^
*T* = 298 K
0.40 × 0.30 × 0.20 mm

#### Data collection

Enraf–Nonius CAD-4 diffractometer
Absorption correction: ψ scan (North *et al.*, 1968 ▶) *T*_min_ = 0.962, *T*_max_ = 0.981
3258 measured reflections
3129 independent reflections
2033 reflections with *I* > 2σ(*I*)
*R*_int_ = 0.029
3 standard reflections every 200 reflections intensity decay: none

#### Refinement

*R*[*F* ^2^ > 2σ(*F* ^2^)] = 0.073
*wR*(*F* ^2^) = 0.197
*S* = 1.02
3129 reflections
235 parameters
H-atom parameters constrained
Δρ_max_ = 0.29 e Å^−3^
Δρ_min_ = −0.27 e Å^−3^

### 

Data collection: *CAD-4 Software* (Enraf–Nonius, 1989 ▶); cell refinement: *CAD-4 Software* ; data reduction: *XCAD4* (Harms & Wocadlo, 1995 ▶); program(s) used to solve structure: *SHELXS97* (Sheldrick, 2008 ▶); program(s) used to refine structure: *SHELXL97* (Sheldrick, 2008 ▶); molecular graphics: *SHELXTL* (Sheldrick, 2008 ▶); software used to prepare material for publication: *SHELXL97*.

## Supplementary Material

Crystal structure: contains datablocks global, I. DOI: 10.1107/S1600536809008484/bq2111sup1.cif

Structure factors: contains datablocks I. DOI: 10.1107/S1600536809008484/bq2111Isup2.hkl

Additional supplementary materials:  crystallographic information; 3D view; checkCIF report

## Figures and Tables

**Fig. 1.**
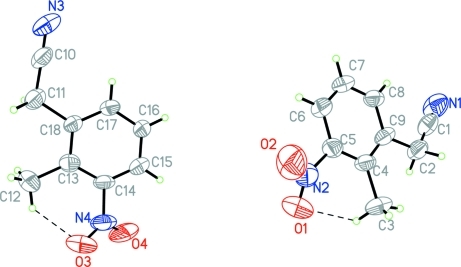
A view of the molecular structure of (I), which is a asymmetric unit with two molecules, with the atom-labeling scheme. Displacement ellipsoids at the 30% probability level. Dash lines indicate C—H···O hydrogen bonds.

## supplementary crystallographic information

### Comment

2-Methyl-3-nitrobenzyl cyanide is important chemical material of effective medicines used for Parkinson's disease, which can be useful not only for the treatment of PD, but also for the treatment of RLS. We report here the crystal structure of the title compound, (I), which is of interest to us in the field.

The molecular structure of (I) is shown in Fig.1, where the dash line indicates C—H···O hydrogen bonds (Table 2). The dihedral angle between the two aromatic rings of the molecules in the asymmetric unit is 20.15 (7)°.

### Experimental

The title compound, (I) was synthesized according to a literature reported before (Wang, 1999). The crystals were obtained by dissolving (I) (0.35 g, 2.0 mmol) into 25 ml of methanol and evaporating the solvent slowly at room temperature for about 4 d.

### Refinement

All H atoms bonded to the C atoms were placed geometrically at the distances of 0.93–0.97Å and included in the refinement in riding motion approximation with *U*_iso_(H) = 1.2*U*_eq_ of the carrier atom. The O—H and N—H distances were constrained to 0.82Å and 0.86Å and these H atoms were refined as riding, with *U*_iso_(H) = 1.2U_eq(_N) and 1.5*U*_eq_(O).

### Crystal data

**Table:**

C_9_H_8_N_2_O_2_	*F*(000) = 736
*M**_r_* = 176.18	*D*_x_ = 1.348 Mg m^−^^3^
Monoclinic, *P*2_1_/*c*	Mo *K*α radiation, λ = 0.71073 Å
Hall symbol: -P 2ybc	Cell parameters from 25 reflections
*a* = 17.216 (3) Å	θ = 10–13°
*b* = 7.1950 (14) Å	µ = 0.10 mm^−^^1^
*c* = 15.746 (3) Å	*T* = 298 K
β = 117.10 (3)°	Block, yellow
*V* = 1736.3 (7) Å^3^	0.40 × 0.30 × 0.20 mm
*Z* = 8	

### Data collection

**Table:**

Enraf–Nonius CAD-4 diffractometer	2033 reflections with *I* > 2σ(*I*)
Radiation source: fine-focus sealed tube	*R*_int_ = 0.029
graphite	θ_max_ = 25.2°, θ_min_ = 1.3°
ω/2θ scans	*h* = −20→18
Absorption correction: ψ scan (North *et al.*, 1968)	*k* = 0→8
*T*_min_ = 0.962, *T*_max_ = 0.981	*l* = 0→18
3258 measured reflections	3 standard reflections every 200 reflections
3129 independent reflections	intensity decay: none

### Refinement

**Table:**

Refinement on *F*^2^	Primary atom site location: structure-invariant direct methods
Least-squares matrix: full	Secondary atom site location: difference Fourier map
*R*[*F*^2^ > 2σ(*F*^2^)] = 0.073	Hydrogen site location: inferred from neighbouring sites
*wR*(*F*^2^) = 0.197	H-atom parameters constrained
*S* = 1.02	*w* = 1/[σ^2^(*F*_o_^2^) + (0.06*P*)^2^ + 2.6*P*] where *P* = (*F*_o_^2^ + 2*F*_c_^2^)/3
3129 reflections	(Δ/σ)_max_ < 0.001
235 parameters	Δρ_max_ = 0.29 e Å^−^^3^
0 restraints	Δρ_min_ = −0.27 e Å^−^^3^

### Special details

**Table:**

Geometry. All e.s.d.'s (except the e.s.d. in the dihedral angle between two l.s. planes) are estimated using the full covariance matrix. The cell e.s.d.'s are taken into account individually in the estimation of e.s.d.'s in distances, angles and torsion angles; correlations between e.s.d.'s in cell parameters are only used when they are defined by crystal symmetry. An approximate (isotropic) treatment of cell e.s.d.'s is used for estimating e.s.d.'s involving l.s. planes.
Refinement. Refinement of *F*^2^ against ALL reflections. The weighted *R*-factor *wR* and goodness of fit *S* are based on *F*^2^, conventional *R*-factors *R* are based on *F*, with *F* set to zero for negative *F*^2^. The threshold expression of *F*^2^ > σ(*F*^2^) is used only for calculating *R*-factors(gt) *etc*. and is not relevant to the choice of reflections for refinement. *R*-factors based on *F*^2^ are statistically about twice as large as those based on *F*, and *R*- factors based on ALL data will be even larger.

### Fractional atomic coordinates and isotropic or equivalent isotropic displacement parameters (Å^2^)

**Table:**

	*x*	*y*	*z*	*U*_iso_*/*U*_eq_	
O1	0.4276 (3)	0.7334 (6)	0.1977 (2)	0.1103 (13)	
O2	0.3421 (2)	0.9322 (6)	0.0964 (3)	0.1045 (12)	
N1	0.7122 (2)	0.5898 (7)	−0.0502 (3)	0.0945 (14)	
N2	0.4027 (3)	0.8202 (6)	0.1242 (3)	0.0745 (10)	
C1	0.6910 (2)	0.6447 (6)	0.0045 (3)	0.0697 (11)	
C2	0.6654 (2)	0.7139 (6)	0.0738 (3)	0.0612 (10)	
H2A	0.6923	0.8344	0.0959	0.073*	
H2B	0.6878	0.6306	0.1282	0.073*	
C3	0.5958 (3)	0.7856 (7)	0.2075 (2)	0.0795 (13)	
H3A	0.6548	0.7639	0.2189	0.119*	
H3B	0.5801	0.6965	0.2425	0.119*	
H3C	0.5910	0.9088	0.2281	0.119*	
C4	0.5348 (2)	0.7660 (5)	0.1016 (2)	0.0516 (9)	
C5	0.4440 (2)	0.7870 (5)	0.0633 (2)	0.0497 (8)	
C6	0.3884 (2)	0.7784 (5)	−0.0346 (2)	0.0552 (9)	
H6A	0.3287	0.7966	−0.0579	0.066*	
C7	0.4230 (2)	0.7427 (5)	−0.0959 (2)	0.0576 (9)	
H7A	0.3867	0.7327	−0.1611	0.069*	
C8	0.5115 (2)	0.7220 (5)	−0.0609 (2)	0.0511 (8)	
H8A	0.5348	0.7000	−0.1029	0.061*	
C9	0.5673 (2)	0.7334 (5)	0.0369 (2)	0.0517 (8)	
O3	0.0637 (2)	0.2551 (6)	0.2586 (2)	0.1082 (13)	
O4	0.1521 (2)	0.0640 (5)	0.2452 (2)	0.1002 (12)	
N3	−0.2116 (2)	0.4233 (6)	−0.2708 (3)	0.0882 (12)	
N4	0.0918 (2)	0.1720 (6)	0.2117 (2)	0.0720 (10)	
C10	−0.1920 (2)	0.3586 (6)	−0.1986 (3)	0.0605 (10)	
C11	−0.1684 (2)	0.2786 (6)	−0.1044 (2)	0.0602 (10)	
H11A	−0.1940	0.1558	−0.1126	0.072*	
H11B	−0.1932	0.3550	−0.0721	0.072*	
C12	−0.1011 (3)	0.1927 (6)	0.0956 (3)	0.0706 (11)	
H12A	−0.0745	0.1150	0.1511	0.106*	
H12B	−0.1529	0.1338	0.0487	0.106*	
H12C	−0.1160	0.3105	0.1129	0.106*	
C13	−0.0386 (2)	0.2224 (5)	0.0551 (2)	0.0495 (8)	
C14	0.0515 (2)	0.2114 (5)	0.1080 (2)	0.0512 (9)	
C15	0.1095 (2)	0.2325 (5)	0.0709 (2)	0.0516 (8)	
H15A	0.1693	0.2203	0.1094	0.062*	
C16	0.0764 (2)	0.2721 (5)	−0.0249 (2)	0.0504 (8)	
H16A	0.1139	0.2890	−0.0520	0.060*	
C17	−0.0121 (2)	0.2867 (5)	−0.0803 (2)	0.0502 (8)	
H17A	−0.0339	0.3126	−0.1450	0.060*	
C18	−0.0704 (2)	0.2634 (5)	−0.0415 (2)	0.0458 (8)	

### Atomic displacement parameters (Å^2^)

**Table:**

	*U*^11^	*U*^22^	*U*^33^	*U*^12^	*U*^13^	*U*^23^
O1	0.128 (3)	0.158 (4)	0.0600 (19)	−0.017 (3)	0.055 (2)	−0.003 (2)
O2	0.086 (2)	0.132 (3)	0.116 (3)	0.003 (2)	0.063 (2)	−0.023 (2)
N1	0.055 (2)	0.133 (4)	0.092 (3)	0.008 (2)	0.030 (2)	−0.020 (3)
N2	0.071 (2)	0.097 (3)	0.060 (2)	−0.022 (2)	0.0345 (19)	−0.020 (2)
C1	0.0400 (19)	0.083 (3)	0.071 (3)	−0.003 (2)	0.0123 (19)	−0.007 (2)
C2	0.0457 (19)	0.075 (3)	0.053 (2)	−0.0042 (18)	0.0141 (17)	−0.0080 (19)
C3	0.075 (3)	0.109 (4)	0.0365 (19)	−0.012 (3)	0.0092 (18)	−0.013 (2)
C4	0.058 (2)	0.054 (2)	0.0315 (16)	−0.0093 (17)	0.0113 (15)	−0.0027 (15)
C5	0.058 (2)	0.0488 (19)	0.0431 (18)	−0.0086 (16)	0.0241 (16)	−0.0080 (16)
C6	0.0438 (18)	0.064 (2)	0.0459 (19)	−0.0013 (17)	0.0100 (15)	0.0042 (17)
C7	0.0483 (19)	0.072 (3)	0.0361 (17)	0.0021 (18)	0.0046 (15)	0.0037 (17)
C8	0.0519 (19)	0.063 (2)	0.0360 (17)	−0.0003 (17)	0.0175 (15)	−0.0011 (16)
C9	0.0432 (18)	0.054 (2)	0.0405 (17)	−0.0022 (16)	0.0039 (14)	−0.0031 (16)
O3	0.122 (3)	0.158 (4)	0.0441 (16)	−0.009 (3)	0.0369 (18)	−0.017 (2)
O4	0.093 (2)	0.117 (3)	0.0479 (17)	0.011 (2)	−0.0052 (16)	0.0230 (18)
N3	0.059 (2)	0.116 (3)	0.056 (2)	0.012 (2)	−0.0029 (17)	0.012 (2)
N4	0.080 (2)	0.088 (3)	0.0327 (16)	−0.010 (2)	0.0124 (17)	−0.0016 (18)
C10	0.0376 (18)	0.074 (3)	0.051 (2)	0.0045 (18)	0.0044 (16)	−0.001 (2)
C11	0.0456 (19)	0.072 (3)	0.052 (2)	0.0026 (18)	0.0125 (16)	−0.0015 (19)
C12	0.072 (3)	0.091 (3)	0.060 (2)	−0.009 (2)	0.041 (2)	0.001 (2)
C13	0.056 (2)	0.0479 (19)	0.0421 (18)	−0.0011 (16)	0.0200 (16)	−0.0057 (15)
C14	0.057 (2)	0.062 (2)	0.0262 (15)	−0.0049 (17)	0.0112 (14)	0.0005 (15)
C15	0.0441 (18)	0.056 (2)	0.0408 (17)	−0.0026 (16)	0.0069 (15)	−0.0022 (16)
C16	0.0410 (17)	0.067 (2)	0.0411 (18)	−0.0052 (16)	0.0167 (14)	−0.0049 (17)
C17	0.0472 (18)	0.063 (2)	0.0314 (16)	−0.0031 (16)	0.0099 (14)	0.0006 (15)
C18	0.0391 (16)	0.055 (2)	0.0357 (16)	−0.0007 (15)	0.0103 (14)	−0.0028 (15)

### Geometric parameters (Å, °)

**Table:**

O1—N2	1.209 (5)	O3—N4	1.209 (5)
O2—N2	1.231 (5)	O4—N4	1.209 (5)
N1—C1	1.148 (5)	N3—C10	1.129 (5)
N2—C5	1.451 (5)	N4—C14	1.483 (4)
C1—C2	1.440 (6)	C10—C11	1.465 (5)
C2—C9	1.522 (5)	C11—C18	1.521 (4)
C2—H2A	0.9700	C11—H11A	0.9700
C2—H2B	0.9700	C11—H11B	0.9700
C3—C4	1.519 (5)	C12—C13	1.492 (5)
C3—H3A	0.9600	C12—H12A	0.9600
C3—H3B	0.9600	C12—H12B	0.9600
C3—H3C	0.9600	C12—H12C	0.9600
C4—C9	1.388 (5)	C13—C14	1.389 (5)
C4—C5	1.404 (5)	C13—C18	1.394 (4)
C5—C6	1.397 (5)	C14—C15	1.375 (5)
C6—C7	1.370 (5)	C15—C16	1.378 (4)
C6—H6A	0.9300	C15—H15A	0.9300
C7—C8	1.371 (5)	C16—C17	1.372 (4)
C7—H7A	0.9300	C16—H16A	0.9300
C8—C9	1.396 (4)	C17—C18	1.402 (4)
C8—H8A	0.9300	C17—H17A	0.9300
			
O1—N2—O2	123.5 (4)	O4—N4—O3	123.7 (4)
O1—N2—C5	118.8 (4)	O4—N4—C14	118.8 (4)
O2—N2—C5	117.7 (4)	O3—N4—C14	117.5 (4)
N1—C1—C2	179.4 (4)	N3—C10—C11	178.2 (4)
C1—C2—C9	114.4 (3)	C10—C11—C18	113.6 (3)
C1—C2—H2A	108.7	C10—C11—H11A	108.8
C9—C2—H2A	108.7	C18—C11—H11A	108.8
C1—C2—H2B	108.7	C10—C11—H11B	108.8
C9—C2—H2B	108.7	C18—C11—H11B	108.8
H2A—C2—H2B	107.6	H11A—C11—H11B	107.7
C4—C3—H3A	109.5	C13—C12—H12A	109.5
C4—C3—H3B	109.5	C13—C12—H12B	109.5
H3A—C3—H3B	109.5	H12A—C12—H12B	109.5
C4—C3—H3C	109.5	C13—C12—H12C	109.5
H3A—C3—H3C	109.5	H12A—C12—H12C	109.5
H3B—C3—H3C	109.5	H12B—C12—H12C	109.5
C9—C4—C5	116.4 (3)	C14—C13—C18	116.1 (3)
C9—C4—C3	120.9 (3)	C14—C13—C12	124.2 (3)
C5—C4—C3	122.6 (3)	C18—C13—C12	119.7 (3)
C6—C5—C4	122.6 (3)	C15—C14—C13	124.6 (3)
C6—C5—N2	116.2 (3)	C15—C14—N4	115.0 (3)
C4—C5—N2	121.2 (3)	C13—C14—N4	120.3 (3)
C7—C6—C5	119.1 (3)	C14—C15—C16	117.9 (3)
C7—C6—H6A	120.5	C14—C15—H15A	121.0
C5—C6—H6A	120.5	C16—C15—H15A	121.0
C6—C7—C8	119.8 (3)	C17—C16—C15	119.8 (3)
C6—C7—H7A	120.1	C17—C16—H16A	120.1
C8—C7—H7A	120.1	C15—C16—H16A	120.1
C7—C8—C9	121.1 (3)	C16—C17—C18	121.5 (3)
C7—C8—H8A	119.4	C16—C17—H17A	119.3
C9—C8—H8A	119.4	C18—C17—H17A	119.3
C4—C9—C8	121.0 (3)	C13—C18—C17	119.9 (3)
C4—C9—C2	118.9 (3)	C13—C18—C11	119.5 (3)
C8—C9—C2	120.1 (3)	C17—C18—C11	120.6 (3)
			
N1—C1—C2—C9	173 (100)	N3—C10—C11—C18	−131 (15)
C9—C4—C5—C6	−0.8 (5)	C18—C13—C14—C15	−2.0 (5)
C3—C4—C5—C6	176.6 (4)	C12—C13—C14—C15	177.8 (4)
C9—C4—C5—N2	179.5 (3)	C18—C13—C14—N4	178.7 (3)
C3—C4—C5—N2	−3.0 (6)	C12—C13—C14—N4	−1.2 (5)
O1—N2—C5—C6	138.3 (4)	O4—N4—C14—C15	−41.7 (5)
O2—N2—C5—C6	−39.6 (5)	O3—N4—C14—C15	136.1 (4)
O1—N2—C5—C4	−42.1 (5)	O4—N4—C14—C13	137.5 (4)
O2—N2—C5—C4	140.0 (4)	O3—N4—C14—C13	−44.7 (5)
C4—C5—C6—C7	1.9 (6)	C13—C14—C15—C16	1.8 (6)
N2—C5—C6—C7	−178.4 (4)	N4—C14—C15—C16	−179.0 (3)
C5—C6—C7—C8	−2.1 (6)	C14—C15—C16—C17	−0.9 (5)
C6—C7—C8—C9	1.1 (6)	C15—C16—C17—C18	0.5 (5)
C5—C4—C9—C8	−0.2 (5)	C14—C13—C18—C17	1.4 (5)
C3—C4—C9—C8	−177.7 (4)	C12—C13—C18—C17	−178.2 (3)
C5—C4—C9—C2	178.6 (3)	C14—C13—C18—C11	−179.8 (3)
C3—C4—C9—C2	1.1 (5)	C12—C13—C18—C11	0.6 (5)
C7—C8—C9—C4	0.0 (6)	C16—C17—C18—C13	−0.7 (5)
C7—C8—C9—C2	−178.7 (3)	C16—C17—C18—C11	−179.5 (3)
C1—C2—C9—C4	168.4 (4)	C10—C11—C18—C13	168.2 (3)
C1—C2—C9—C8	−12.9 (5)	C10—C11—C18—C17	−13.0 (5)

### Hydrogen-bond geometry (Å, °)

**Table:**

*D*—H···*A*	*D*—H	H···*A*	*D*···*A*	*D*—H···*A*
C3—H3B···O1	0.96	2.40	2.852 (8)	108
C12—H12A···O3	0.96	2.42	2.864 (6)	108
